# Editorial: IgA nephropathy: a nephrologist’s challenge in 2023

**DOI:** 10.3389/fneph.2024.1477350

**Published:** 2024-09-11

**Authors:** Fausta Catapano, Oliver Flossmann, Lucia Del Vecchio

**Affiliations:** ^1^ Nephrology, Dialysis and Hypertension Unit, IRCCS Azienda Ospedaliero-Universitaria di Bologna, Bologna, Italy; ^2^ Berkshire Kidney Unit, Royal Berkshire Hospital, Reading, United Kingdom; ^3^ Department of Nephrology and Dialysis, ASST Lariana, San Fermo della Battaglia, Italy

**Keywords:** IgA nephropathy, treatment, pathogenesis, cathepsin S (Cat S), BAFF - B-cell activating factor, APRIL (TNFSF13), biomarker, SGLT 2 inhibitor

IgA nephropathy (IgAN) is the most common primary glomerulonephritis in young people worldwide. It is characterized by mesangial deposition of immune-complexes containing galactose-deficient-IgA1 and associated autoantibodies; thus, it is now considered an autoimmune disease. The prevalence of IgAN is higher in Asian compared to European and sub-Saharan African populations, due to genetic and environmental factors and to the differences in the frequency of urine screening and threshold for performing kidney biopsy, the gold standard for the diagnosis. IgAN accounts for approximately 40% of all native biopsies in Japan, 25% in Europe, 12% in USA, and <5% in Central Africa (1).

Although slowly, end-stage kidney disease occurs in 25%–50% of patients within 10–20 years of presentation (2–4). Thus, many young patients will start dialysis in their lifetime, even when treated with currently available therapy (4). In older patients, IgAN can increase cardiovascular risk and anticipate initiation of dialysis (5). Broadening the criteria for kidney biopsy would allow earlier diagnosis and treatment, possibly improving this outcome. The Oxford Classification has shown the prognostic importance of histological lesions, and a recent Chinese study assumes that the intensity of glomerular macrophage invasion can predict the response to immune-suppressive therapy (6). Thus, repeat biopsies may become important if increased disease activity is clinically suspected.

Hypertension and proteinuria are independent risk factors for disease progression in IgAN (3, 7). Current and main therapeutic goals are proteinuria <1 g/day, better if <0.5 g/day (8, 9), it is then reasonable to assume that reducing proteinuria as low and as early as possible is worthwhile; and blood pressure <120/80 mmHg, as Kidney Disease: Improving Global Outcomes (KDIGO) guidelines recommend (10).

Angiotensin-converting-enzyme inhibitors (ACE inhibitors) or Angiotensin receptor blockers (ARB) are the first choice at maximum tolerated doses, irrespective of hypertension (10). Lifestyle changes, in particular dietary sodium restriction, weight control, and aerobic exercise, are essential parts of IgAN treatment (10). Thus, encouraging the patient to adopt a healthy lifestyle becomes part of the management as well as its active participation in treatment decisions.

This Research Topic arises from the *ferment* in IgAN research. Advances in the understanding of pathogenesis (GWAS studies), disease assessment, and international collaboration have led to new medications approved by FDA and EMA, providing hope that future treatment options will become increasingly precise and personalized.

According to the “four-hit hypothesis,” elevated circulating IgA1 levels that lack galactose residues (Gd-IgA1) (hit 1), in susceptible individuals, trigger autoantibody production (hit 2) that causes formation (hit 3) and deposition of immune-complexes in the mesangium, triggering the complement cascade, cellular proliferation, and cytokine and chemokine release and leading to kidney injury in IgAN (hit 4) (11). In a review by Cheung et al., the pivotal roles of BAFF (B-cell-activating factor of the TNF family) and APRIL (A proliferation-inducing ligand) in IgAN pathogenesis were explored, in particular in their interplay between mucosal hyper-responsiveness (Human gut-associated lymphoid tissues (GALT) and nasal-associated lymphoid tissue (NALT)) and B-cell activation with overproduction of Gd-IgA1 and its autoantibodies. Preclinical and clinical studies demonstrate the relationship between high levels of both mediators and IgA levels. As BAFF is primarily involved in the survival and maturation of B cells (12) and APRIL in the later stages of B-cell differentiation (13), their single or dual inhibition is to be considered as a therapeutic strategy. Sibeprenlimab, an anti-APRIL-humanized antibody (14), telitacicept and atacicept, two novel agents that target both BAFF and APRIL tested in Chinese IgAN patients (15, 16), have shown promising results.

In their original research, Fu et al., through a two-sample Mendelian randomization, observed a causal relationship between elevated cathepsin S (CTSS) levels and increased risk of developing IgAN. Cathepsins are a class of enzymes regulating protein trafficking and secretion in the proteolytic events of many kidney and autoimmune diseases (17–21). As they also found higher serum CTSS and higher CTSS expression in tubulo-interstitium of IgAN patients compared to controls and other primary glomerulonephritis, they hypothesized that CTSS could influence immune-mediated processes and metabolic pathways in IgAN. They also observed that CTSS expression positively correlate with monocytes and T cells gamma delta infiltration in IgAN renal tissues. Thus, they suggested that CTSS could be an exciting drug target for IgAN treatment.

Noor et al. presented an overview of available and eligible drugs for IgAN. They discussed two new drugs approved by FDA: TRF-budesonide and sparsentan. TRF-budesonide acts on Peyer’s patches, the primary site for IgA production, and minimizes systemic exposure to steroids. In the NefIgArd trial, patients treated with maximum Renin-angiotensin-aldosterone system (RAAS) inhibition and targeted-release formulation (TRF)-budesonide for 9 months had a 48% reduction in UPCR (22) and eGFR over 2 years was higher in TRF-budesonide compared to placebo, even after 15 months of drug discontinuation (23). Sparsentan, an oral dual endothelin and angiotensin II-receptor antagonist, has been approved for high-risk IgAN patients. In the PROTECT trial, urine protein-to-creatinine ratio (UPCR) was reduced by 49.8% in the sparsentan group compared with the irbesartan group at 36 weeks (24). Keeping in mind the side effects of steroids, in IgAN patients at high risk of progression (higher proteinuria, eGFR loss, and MEST-C score), low-dose steroids, as in the TESTING 2.0 trial, should be considered, whereas clinical trial enrollment is desirable. Sodium-glucose cotransporter 2 (SGLT-2) inhibitors have been approved for chronic kidney disease; through the reduction of intraglomerular pressure by tubulo-glomerular feedback, they have proven to be effective in reducing UPCR by 26% in IgAN (25) and slowing the rate of progression of chronic kidney disease in all patients, including those with low-grade albuminuria (26). There are ongoing studies on other endothelin-receptor antagonists and non-steroidal anti-aldosteron antagonists, alone or in combination with i-SGLT-2.

Yoshimura et al. presented a 51-year-old male patient affected by alcoholic liver cirrhosis (ALC) with renal impairment (serum creatinine: 2 mg/dL) and nephrotic syndrome (UPCR 4.3 g/gCr) due to biopsy-proven IgAN. Dapagliflozin (10 mg/day) was initiated, and surprisingly, after 1 week of treatment, UPCR was markedly reduced to <1 g/gCr and hematuria disappeared. The authors assumed a potential efficacy of iSGLT-2 for renal protection in IgAN-ALC. Since in DAPA-CKD and EMPA-KIDNEY nephrotic syndrome was only slightly represented, caution is needed to use SGLT-2 inhibitors in these patients.

We thank the contributing authors for demonstrating interest in this Research Topic and for sharing their research, while waiting for the results of ongoing trials and the new KDIGO guidelines for IgA nephropathy.
